# Physical Activity Earlier in Life Is Inversely Associated With Insulin Resistance Among Adults in Japan

**DOI:** 10.2188/jea.JE20170180

**Published:** 2019-02-05

**Authors:** Hitomi Fujita, Akihiro Hosono, Kiyoshi Shibata, Shoko Tsujimura, Kyoko Oka, Naoko Okamoto, Mayumi Kamiya, Fumi Kondo, Ryozo Wakabayashi, Mari Ichikawa, Tamaki Yamada, Sadao Suzuki

**Affiliations:** 1 Department of Public Health, Nagoya City University Graduate School of Medical Sciences, Nagoya, Japan; 2 Aichi Gakusen University, Aichi, Japan; 3 Okazaki City Medical Association, Public Health Center, Aichi, Japan

**Keywords:** physical activity, insulin resistance, HOMA-IR, diabetes mellitus, lifestyle related diseases

## Abstract

**Background:**

It is known that physical activity affects glucose metabolism. However, there have been no reports on the influence of physical activity earlier in life on subsequent glucose metabolism. Therefore, we analyzed the influence of physical activity in earlier decades of life on insulin resistance in middle aged and older residents in Japan.

**Methods:**

The subjects were 6,883 residents of Okazaki City between the ages of 40 and 79 years who underwent physical examinations at the Okazaki City Medical Association Public Health Center from April 2007 through August 2011. They gave informed consent for participation in the study. Data on individual characteristics were collected via a questionnaire and from the health examination records. Fasting blood glucose and insulin levels were used to calculate the homeostatic model assessment of insulin resistance (HOMA-IR). HOMA-IR >1.6 was considered to indicate insulin resistance for the purpose of logistic regression models.

**Results:**

The study sample included 3,683 men and 3,200 women for whom complete information was available. For those who exercised regularly throughout their teens to their 30s–40s, the odds ratio for having insulin resistance was 0.75 (95% confidence interval [CI], 0.58–0.96) for men and 0.76 (95% CI, 0.58–0.99) for women after adjusting for other variables, including age, body mass index, and present physical activity. A linear trend was also observed in both men and women.

**Conclusions:**

Subjects who have exercised regularly in the early decades of life are less likely to have insulin resistance later in life.

## INTRODUCTION

It is well-known that people who are physically active are at a lower risk of mortality and morbidity related to ischemic heart disease, hypertension, diabetes, obesity, and osteoporosis.^1^^,^^2^ Physical activity is also beneficial to mental health and quality of life.^3^ Despite the variety of benefits that physical activity brings, however, there are very few long-term chronological studies on the effects of physical activity on subsequent prevention of lifestyle-related diseases.^4^ The prevalence of such diseases is increasing,^5^ and more specific diagnostic and preventive measures are required.^6^ An insulin resistance index has been widely used to detect signs of type 2 diabetes mellitus at an early stage.^4^^,^^6^ Physical activity is known to decrease insulin resistance.^7^ There are few reports, however, on the long-term effects of physical activity on glucose metabolism. Thus, we decided to investigate the association of physical activity in the early decades of life with insulin resistance among middle-aged and older individuals.

## METHODS

### Study population

The present study was conducted in Okazaki City, Aichi Prefecture, Japan, from April 2007 through August 2011 as a part of the Japan multi-institutional collaborative cohort (J-MICC) study. Study participants were selected from residents of Okazaki City aged 40 to 79 years on the day they registered for a health checkup at the Okazaki Public Health Center. An invitation letter and an accompanying questionnaire were mailed from the Health Center prior to the examination to examinees who were eligible for the study.

All participants were asked to read and sign a detailed informed consent document. A total of 7,493 examinees (4,139 men and 3,354 women) agreed to participate. The study protocol of the Okazaki study was approved by the university institutional review board (Nagoya City University Graduate School of Medical Sciences and Medical School).

### Baseline data and measurements

Data on sex, age, present physical activity, physical activity in earlier decades of life, smoking and drinking habits, and use of hypoglycemic drugs was collected from the questionnaire. Fasting blood glucose (FBG; in mmol/L) and insulin (µU/mL) levels were used to calculate the homeostatic model assessment of insulin resistance (HOMA-IR), an index of insulin resistance (HOMA-IR = FBG × insulin/22.5).^8^ The Japan Diabetes Society has recommended HOMA-IR ≤1.6 as indicating non-IR and HOMA-IR ≥2.5 as identifying IR in their “Treatment Guide for Diabetes 2016”.^9^ In the present study, we used threshold of HOMA-IR ≤1.6.

In regard to past physical activity, participants were asked “How often did you exercise when you were in your teens, 20s, and 30–40s?”. Possible answers were: 1) none, 2) almost none, 3) some, 4) a lot, and 5) too much. Answers 1) and 2) were categorized as “(−) no exercise” and answers 3) to 5) as “(+) having exercised” for each decade. Next, participants were separated into three groups for analysis: Consistent, those who exercised in all decades; Some, those who exercised in one or two decades; and None, those who did not exercise at any age (Table 1). Subjects were further classified based on present physical activity: “yes,” those whose present physical activity included both walking one hour or more a day and ≥30 minutes of exercise once a week; “some,” those who did either of the above activities; and “none,” those who did neither.

**Table 1.** Classification by past physical activity

	Past physical activity in teens			
	−: none or almost none		+: some, a lot or too much	
	
	Past physical activity in 30–40s		Past physical activity in 30–40s	
	−	+	−	+
Past physical activity in 20s				
−: none or almost none	None	Sometimes	Sometimes	Sometimes
+: some, a lot, too much	Sometimes	Sometimes	Sometimes	Consecutively

Subjects with insufficient data (24 men and 22 women), who used hypoglycemic drugs (353 men and 117 women), or whose FBG was >7.78 mmol/L (79 men and 15 women) were excluded.

### Statistical analysis

In order to determine the relationship of past physical activity with insulin resistance among adults, we performed logistic regression analysis to adjust for age (continuous), body mass index (BMI; continuous), alcohol drinking (present, past, or none), smoking habits (present, past, or none), and present physical activity habits (yes, some, or none). Statistical analyses were performed with EZR (ver. 1.3.3: Saitama Medical Center, Jichi Medical University, Saitama, Japan) and men and women were analyzed separately.^10^ All of the *P* values were two-sided, with *P* < 0.05 indicating statistical significance.

## RESULTS

After applying the exclusion criteria, data from a total of 3,683 men and 3,200 women were analyzed. Table 2 classifies subjects by past physical activity and reports the numbers or mean values of various factors. Men and women responding with “some” exercise in the past composed the largest group and had the youngest average age. Those whose exercise was “consistent” through earlier decades had the highest percentage of individuals who maintained physical activity in the present, approximately 10% to 15% higher than “some” or “none” among both men and women.

**Table 2.** Characteristics of study participants according to past physical activity

Characteristics	Men (*n* = 3,683)						Women (*n* = 3,200)					
	
	Past physical activity						Past physical activity					
	
	None		Sometimes		Consecutively		None		Sometimes		Consecutively	
Number, *n* (row%)	515	(14.0%)	1700	(46.2%)	1468	(39.9%)	775	(24.2%)	1739	(54.3%)	686	(21.4%)
Age, years, mean (SD)	60.4	(9.8)	55.9	(11.3)	62.0	(9.7)	57.7	(9.4)	54.5	(10.6)	59.2	(10.2)
HOMA-IR, mean (SD)	1.40	(1.0)	1.51	(1.0)	1.33	(0.9)	1.31	(0.8)	1.29	(0.8)	1.31	(0.9)
HOMA-IR >1.6, *n* (row%)	154	(29.9%)	578	(34.0%)	383	(26.1%)	206	(26.6%)	415	(23.9%)	169	(24.6%)
Insulin, µU/mL, mean (SD)	5.68	(3.7)	6.07	(3.8)	5.37	(3.2)	5.57	(3.0)	5.48	(3.1)	5.52	(3.2)
Fasting blood glucose, mmol/L, mean (SD)	5.46	(0.56)	5.48	(0.59)	5.48	(0.57)	5.23	(0.52)	5.19	(0.51)	5.22	(0.52)
BMI, kg/m^2^, mean (SD)	23.1	(2.9)	23.6	(3.0)	23.4	(2.8)	22.1	(3.0)	22.1	(3.1)	22.4	(2.9)
Smoking habit, *n* (%)												
current	111	(21.6%)	459	(27.0%)	380	(25.9%)	35	(4.5%)	137	(7.9%)	39	(5.7%)
past	227	(44.1%)	814	(47.9%)	674	(45.9%)	31	(4.0%)	125	(7.2%)	43	(6.3%)
none	177	(34.4%)	427	(25.1%)	414	(28.2%)	709	(91.5%)	1477	(84.9%)	604	(88.0%)
Alcohol drinking, *n* (%)												
current	348	(67.6%)	1239	(72.9%)	1092	(74.4%)	244	(31.5%)	619	(35.6%)	219	(31.9%)
past	19	(3.7%)	56	(3.3%)	41	(2.8%)	12	(1.5%)	34	(2.0%)	14	(2.0%)
none	148	(28.7%)	405	(23.8%)	335	(22.8%)	519	(67.0%)	1086	(62.4%)	453	(66.0%)
Present physical activity, *n* (%)												
yes	169	(32.8%)	519	(30.5%)	650	(44.3%)	251	(32.4%)	557	(32.0%)	291	(42.4%)
somewhat	211	(41.0%)	650	(38.2%)	537	(36.6%)	358	(46.2%)	788	(45.3%)	289	(42.1%)
none	135	(26.2%)	531	(31.2%)	281	(19.1%)	166	(21.4%)	394	(22.7%)	106	(15.5%)
Past physical activity, *n*												
teens	0		1483		1468		0		1433		686	
20s	0		829		1468		0		537		686	
30s	0		288		1468		0		561		686	

BMI, body mass index; HOMA-IR, homeostatic model assessment of insulin resistance; SD, standard deviation.

Table 3 shows insulin resistance status and the odds ratios for insulin resistance in relationship to past physical activity, with the “none” group as the reference. The lowest odds ratios were observed among those with “consistent” exercise in both men (odds ratio 0.75; 95% confidence interval, 0.58–0.96) and women (odds ratio 0.76; 95% confidence interval, 0.58–0.99), after adjusting for age, BMI, history of drinking alcohol, history of smoking, and present physical activity. A linear trend was also observed for both men and women (men: *P* for trend = 0.0026; women: *P* for trend = 0.047).

**Table 3.** The odds ratio of insulin resistance (HOMA-IR >1.6) associated with past physical activity

Past physical activity	Men					Women				
	
	HOMA-IR >1.6/≤1.6	Age adjusted OR (95% CI)		Multivariate^a^ OR (95% CI)		HOMA-IR >1.6/≤1.6	Age adjusted OR (95% CI)		Multivariate^a^ OR (95% CI)	
None	154/361	1	(Reference)	1	(Reference)	206/569	1	(Reference)	1	(Reference)
Sometimes	578/1122	1.12	(0.91–1.39)	1.02	(0.80–1.31)	415/1324	0.95	(0.78–1.16)	0.93	(0.75–1.16)
Consecutively	383/1085	0.85	(0.68–1.06)	0.75	(0.58–0.96)	169/517	0.86	(0.68–1.09)	0.76	(0.58–0.99)
*P* for trend		0.018		0.0026			0.21		0.047	

BMI, body mass index; CI, confidence interval; HOMA-IR, homeostatic model assessment of insulin resistance; OR, odds ratio.

^a^Adjusted for age, BMI, alcohol drinking, smoking habit and present physical activity.

## DISCUSSION

To the best of our knowledge, this study is the first to focus on the association of early physical activity with insulin resistance later in life. Insulin resistance impacts the metabolism not only of glucose but also of fats, and it is associated with the development of diabetes, high blood pressure, and arteriosclerosis.^11^ Thus, early detection of insulin resistance might contribute to increased efforts to prevent lifestyle-related diseases. Physical activity and physical therapy are positively correlated with a decrease in insulin resistance because the skeletal muscles are insulin’s main target organs.^12^^–^^14^ Furthermore, the effects of physical therapy may bring about long-term changes in the body, in addition to short-term effects.^15^^–^^17^ In this research, we investigated the long-term association between past physical activity and glucose metabolism.

The beneficial association of past physical activity with insulin resistance in this study was not due to weight loss, since past physical activity still had a significant trend toward decreased insulin resistance after adjustment for BMI among both men and women. It was interesting that the group that exercised consistently had a higher BMI than those with no physical activity. The positive association with physical activity, regardless of BMI, was consistent with the findings of Fukushima et al.^18^

The higher BMI we observed among consistent exercisers might be due to increased muscle mass, which is expected to affect insulin resistance.^19^ Insulin functions as a protein anabolic hormone, suppressing degradation of muscle protein and facilitating muscle protein synthesis.^20^ The degree of change in muscle protein synthesis caused by insulin stimulation is inversely correlated with HOMA-IR, and the protein metabolic cycle is stimulated by physical activity.^21^ Furthermore, lipids in skeletal muscle cells can have a significant influence on insulin resistance. As the quantity and quality of intramuscular lipids are controlled by physical activity,^22^^–^^26^ we consider this also to be part of the protein metabolic cycle. Thus, continued physical activity contributes to an increase in capillary density and eventual change in skeletal muscle fiber composition.

We analyzed the relation to physical activity in each specific age period, finding that exercise in each decade was associated with lower odds of insulin resistance among both men and women (data not shown). Several studies have reported the effect of physical activity in a specific period in the past.^27^^,^^28^ Our findings extend these results, supporting the recommendation for physical activity for all ages from the second through the fifth decade of life.

Our study has some limitations that should be considered when interpreting the results. First, as a cross-sectional study, information regarding past physical activity was collected using a questionnaire, so that the results rely on the subjects’ recall. However, it is unlikely that misclassification would occur systematically, as self-evaluation of the past amount of physical activity was done without the subjects’ knowledge of their HOMA-IR. Thus, we think it unlikely that there was information bias or that misclassification of physical activity affected the results. Second, regarding past physical activity, subjects were asked to give their subjective estimate of physical activity rather than actually quantifying it. Self-assessment is often estimated to be lower than actual physical activity.^29^ If there was misclassification on this basis, the odds ratios may have been lower than the true values. Third, those who were physically active from their teens through their 40s are generally likely to remain physically activity after their 50s. Unfortunately, since there is no information on past physical activity since their 50s in this study, these effects cannot be assessed. However, changes in physical activity after their 50s could be evaluated via present physical activity to some extent. Therefore, the effect due to the lack of this information would be limited. Fourth, we lacked data on diet in this study and could not assess the influence of diet on physical activity. However, we believe that the influence of diet can be summarized using BMI. In addition, the threshold of insulin resistance should be mentioned. The threshold of HOMA-IR for insulin resistance is controversial even among Japanese studies, which have used cut-off values between 1.7^30^ and 2.0.^31^ The threshold of 1.6 was based on the Japan Diabetes Society recommendation (<1.6 was indicating non-IR). When we used the threshold of 2.0, the tendency of the results was similar to the present ones, although less stable because of the small number of cases of insulin resistance (data not shown).

### Conclusion

Past physical activity was inversely associated with present insulin resistance. It is hoped that this study, focusing on the influence of physical activity at an early age on insulin resistance later in life, will be useful in motivating young people to cultivate healthy physical activity habits as an investment for their old age.
